# Molecular Characterization of High-Risk Human Papillomavirus in Women in Bobo-Dioulasso, Burkina Faso

**DOI:** 10.1155/2016/7092583

**Published:** 2016-07-20

**Authors:** Ina Marie Angèle Traore, Théodora Mahoukèdè Zohoncon, Adama Dembele, Florencia W. Djigma, Dorcas Obiri-Yeboah, Germain Traore, Moussa Bambara, Charlemagne Ouedraogo, Yves Traore, Jacques Simpore

**Affiliations:** ^1^Laboratory of Molecular Biology and Genetics (LABIOGENE), University of Ouagadougou, P.O. Box 7021, Ouagadougou, Burkina Faso; ^2^Pietro Annigoni Biomolecular Research Center (CERBA), P.O. Box 364, Ouagadougou, Burkina Faso; ^3^Department of Gynecology, Obstetrics and Reproductive Medicine, Sourou Sanou Teaching Hospital, P.O. Box 676, Bobo-Dioulasso, Burkina Faso; ^4^School of Medical Sciences, Microbiology Department, University of Cape Coast, Cape Coast, Ghana; ^5^Polyvalent Medical Center-Health and Reproduction, P.O. Box 1418, Bobo-Dioulasso, Burkina Faso; ^6^Yalgado Ouédraogo University Hospital (CHU/YO), P.O. Box 7022, Ouagadougou, Burkina Faso; ^7^UFR/SDS, University of Ouagadougou, P.O. Box 7021, Ouagadougou, Burkina Faso; ^8^Training and Research Unit in Life and Earth Science (UFR/SVT), University of Ouagadougou, P.O. Box 7021, Ouagadougou, Burkina Faso; ^9^Faculty of Medicine, University Saint Thomas d'Aquin, P.O. Box 10212, Ouagadougou, Burkina Faso

## Abstract

High-risk human papillomavirus (HPV) is found in over 99% of cervical cancers. The aim of this study was to determine the prevalence of HPV in a population of women in Bobo-Dioulasso and to identify the high-risk types present in these women. From May to June, 2015, 181 women who came for consultation at the Souro Sanou University Hospital of Bobo-Dioulasso have been included in this study. Uterine endocervical swabs have been taken in these women. DNA obtained by extraction from the samples thus collected was used to determine the prevalence of high-risk human papillomavirus genotypes through real-time PCR. The age of the women ranged from 20 to 56 years with a mean of 35.3 ± 8.1 years. The prevalence of infection by high-risk HPV types was 25.4% (46/181). The most common high-risk HPV genotypes were HPV 39 (18.5%), HPV 52 (16.7%), HPV 18 (14.8%), and HPV 35 (13.0%). HPV 16 which is included in the HPV vaccines was not found in the population studied. This type of study which is the first one in Bobo-Dioulasso has showed a high prevalence of genotypes HPV 39, HPV 52, and HPV 35 which are not yet covered by a vaccine.

## 1. Introduction

The human papillomavirus (HPV) infection is one of the most common sexually transmitted infections in the world [1, 2]. In most cases, the infection may be unnoticed after a clearance period between 8 and 12 months [3]. The persistence of HPV infection with high oncogenetic risk is a condition for the occurrence of cervical precancerous and cancerous lesions [4]. When these lesions are not treated, some are likely to progress into cancer and HPV is found in 99.7% of cervical cancers [5]. With an incidence estimated at about 530,000 new cases and 275,000 deaths worldwide each year [6], cervical cancer is the leading cancer in women in sub-Saharan Africa and it remains a serious public health issue. While HPV 16 and HPV 18 genotypes are involved in approximately 70% of cancers of the cervix in the world [7], the distribution of the other genotypes follows geographic variation. This difference in the distribution of genotypes was found in women who were living in different regions of the same country [8]. In Burkina Faso, the study conducted by Zohoncon et al. in 2013 based on two previous studies in Ouagadougou [9, 10] showed that the high-risk HPV genotypes which were the most common were HPV 35 and HPV 52 [11]. In order to determine if the distribution of high-risk HPV genotypes was following the same trend in another city in Burkina Faso, we undertook this study. The aim of this study was to determine the prevalence of high-risk human papillomavirus in a population of women in Bobo-Dioulasso and to identify genotypes found in this city.

## 2. Materials and Methods

The study was conducted at the Souro Sanou University Hospital of Bobo-Dioulasso, the second city of Burkina Faso. From May to June, 2015, 181 women who came for consultation in the Department of Gynecology, Obstetrics, and Reproductive Medicine (DGOMR) have been included in the study. Pregnant women or women who have undergone hysterectomy or menstruating women have been excluded. Each woman answered a questionnaire to provide information on their socioeconomic status and behavioral and sexual habits. Samples have been taken through endocervical swabbing of the uterus using a sterile cotton swab. The samples thus obtained have been immersed in a transport medium which was provided with DNA-Sorb-A kit (Sacace Biotechnologies, Como, Italy) and kept at −20°C until the DNA extraction. Immediately after sampling, screening for precancerous lesions was done for the women by visual inspection with acetic acid and Lugol's iodine (VIA/VILI).

This study has been approved by the Ethics Committee for Research in Health of Burkina Faso (Deliberation number 2014-9-110) and all the women have signed a consent form prior to participation.

The DNA extraction was made using DNA-Sorb-A kit (Sacace Biotechnologies, Como, Italy). The genotyping of high-risk HPV was made by real-time PCR using the kit “HPV Genotypes 14 Real-TM Quant,” code V67-100FRT (Sacace Biotechnologies, Como, Italy), and the Sacycler-96 Real-Time PCR (Sacace Biotechnologies, Como, Italy). This genotyping is based on multiplex real-time amplification for each sample and the *β*-globin gene was used as internal control. The “HPV Genotypes 14 Real-TM Quant” made it possible to detect the following 14 high-risk HPV genotypes: 16, 18, 31, 33, 35, 39, 45, 51, 52, 56, 58, 59, 66, and 68.

The PCR program used was as follows: 1 cycle of 95°C for 15 minutes; 5 cycles of 95°C for 5 seconds, 60°C for 20 seconds, 72°C for 15 seconds; and 40 cycles of 95°C for 5 seconds, 60°C for 30 seconds, and 72°C for 15 seconds.

Data were entered and analyzed using the SPSS software in its 20.0 version and Epi Info 7. The Chi-square test was used for comparisons with a significant difference for *p* < 0.05. Women under 30 years were considered as young women and these criteria were used in the study to group the women in age group: ≥30 years old versus <30 years old.

## 3. Results

During the enrollment period in the Department of Gynecology, Obstetrics, and Reproductive Medicine (DGOMR), 181 women gave their consent to participate to this study. The main motivations for consultation were leucorrhea and pruritus (41, 4%), cervical cancer screening (18, 2%), abdominal-pelvic pain (11, 6%), dysmenorrhea (7, 2%), contraception needs (6, 1%), maternity desire (4, 4%), breast pain (3, 9%), and others (7, 2%).

### 3.1. The Sociodemographic Characteristics of the Women in the Study

The sociodemographic, sexual, and behavioral characteristics of the study population are given in Table 1. The women's age ranged from 20 to 56 years with a mean of 35.3 ± 8.1 years; and 50.8% of the women were under 35. The majority of women (84%) were married or lived with a partner. Housewives and women working in the informal sector accounted for 68.5% and 15.5%, respectively; only 8.8% of the women were employed and 7.2% were either high school students or university students. As for education level, 34.3% and 1.1% of the women had reached high school and university, respectively. The others were either illiterate (35.9%) or had primary level (28.7%). The age of the women at first sexual intercourse ranged from 13 to 27 years with a mean of 18.57 ± 2.2 years. Among all women, only 6.6% had never been pregnant. The other had at least one pregnancy in their lives and 18.8% of them had more than 5 children. More than half of the women (67.4%) had never had an abortion while the others had at least one miscarriage. In addition, 66.3% of the women were not using contraception at the time of inclusion in the study.

### 3.2. The Prevalence of High-Risk HPV Genotypes

**Figure 1:**
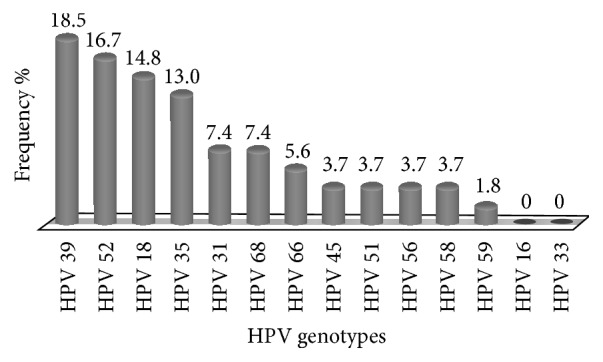
Frequency of high-risk HPV genotypes found among the women in the study.

In this study, all tested samples were positive for *β*-globin gene. The results of HPV research show that 25.4% (46/181) of the women were infected with high-risk HPV. The number of HPV genotypes per woman ranged from 1 to 3, and in considering multiple infections, we counted in total 54 genotypes among the 46 women infected with HPV.  Figure 1 shows the distribution of these genotypes among which the most common ones were HPV 39 (18.5%), HPV 52 (16.7%), HPV 18 (14.8%), and HPV 35 (13.0%). HPV 16 and HPV 33 were not found in the women included in this study.

### 3.3. Distribution of Genotypes Based on the Type of Infection

84.8% of the women infected with HPV were carriers of a single genotype. There was 15.2% (7/46) of multiple infections including 6 multiple infections with 2 genotypes and 1 multiple infection with 3 genotypes (Table 2). This table also shows that the distribution of genotypes found in this study was not statistically different among the women whether they are over or under 30 years of age (*p* = 0.335).

### 3.4. Potential Risk Factors Associated with the Carrying of HPV

In Table 3, we analyzed association between the HPV infection and age, age at first sexual intercourse, parity, the use of oral contraception, and the VIA/VILI result. On the 181 women of the study, visual screening for precancerous lesions was not done for 2 of them. The results showed that 9/179 women or 5.0% were positive to VIA/VILI. This study found no statistical association between HPV infection and the risk factors analyzed.

## 4. Discussion

This study shows the characteristics of the high-risk HPV infection in a general population of women in Bobo-Dioulasso. To the best of our knowledge, in Bobo-Dioulasso, it is the first study on a general population of women concerning the HPV. Indeed, the other studies conducted in this city have focused on a particular group of women who are sex workers [12, 13]. However, the majority of women (90.6%) in the current study had a single sexual partner and 7.2% of the women reported no sexual activity at the time of inclusion in the study. The women in this study had a mean age of 35.3 years; Ouedraogo et al. in 2011 [10] had also found an average age of over 30 years among women attending gynecological consultation in Ouagadougou. But unlike this study which counted 38.3% of housewives, more than half (68.5%) of the women in our study were housewives.

The prevalence of 25.4% of high-risk HPV obtained in this study is high compared to the worldwide prevalence of HPV infection estimated to be around 11-12% [14]. Several studies suggest a higher prevalence of oncogenic HPV type in sub-Saharan Africa compared to elsewhere [15–17] and the average prevalence reported in this part of Africa was 24% [14]. The prevalence of high-risk HPV in this study is in line with the 23.2% reported in Thiès, in Senegal [8], and the 23% in rural women in Mali [18]. In two studies conducted in Ouagadougou, Burkina Faso [11, 19], the high-risk HPV prevalence was 30.2% and 41.5%, higher than which was found in the present study. The last study conducted in Ouagadougou focused on adolescent girls and this could explain the difference in the prevalence compared to this study. Indeed, the average age of our population was 35.3 years while it has been indicated that the prevalence of high-risk HPV infection was maximum before the age of 30 years [20, 21].

HPV 39 (18.5%), HPV 52 (16.7%), HPV 18 (14.8%), and HPV 35 (13.0%) were the most common genotypes in this study. The frequency of the other genotypes found ranged from 7.4 to 1.8%. HPV 39 was not commonly found at the top of the most common genotypes in the literature. But it was part of the 3 most common genotypes in women with Atypical Squamous Cells of Undetermined Significance (ASCUS) [22]. A high prevalence of HPV 52 was also observed in Japan [23], Tanzania [24], and Burkina Faso [11, 19]. In Didelot-Rousseau et al.'s [12] study conducted in Bobo-Dioulasso, HPV 52 was the most frequent type followed by HPV 35. These authors examined both high and low risk HPV types; HPV 16 with HPV 18 were, respectively, the 6th and the 8th most frequent genotype found in women. The prevalence of HPV 18 obtained in the present study was 14.8%; it is greater than that reported in Bobo-Dioulasso (6.4%) [12] and even in Colombia (7.1%) [25]. However, it is less than the value found in South African women (21.0%) [24]; the authors of that study showed a regional difference in the distribution of HPV 18.

HPV 16 and HPV 18 are the high-risk genotypes included in the vaccines available to fight against cervical cancer. HPV 16 was not found in this study. Some studies had already shown a low prevalence of HPV 16 in some African countries compared to other high-risk HPV genotypes [1, 11, 19, 26]. However, HPV 16 was found as the most common genotype in the world especially in Europe [27], the USA [28], and North Africa [29].

In this study, the carrying of the high-risk HPV was not associated with risk factors such as the age at first sexual intercourse before the age of 16 [30], the use of oral contraception, the parity, or the VIA/VILI result. Human Immunodeficiency Virus (HIV) infection may also be considered as a risk factor that influences HPV infection. Indeed, HIV upregulates HPV replication [31] and HPV persistence [32] leading to the evolution of HPV infection from high-risk lesions to uterine cervix cancer. It is known that HIV-positive women have higher proportion of HPV infection than HIV-negative and HIV infection could have an impact on HPV genotype distribution [33].

In the present study, we did not investigate women's HIV status but it is known that HIV prevalence for the general population of Bobo-Dioulasso is 1.4% [34]. This value is within the highest prevalence of Burkina Faso where adults HIV prevalence is estimated to be less than 1% [35]. The relative high HIV prevalence for the general population of Bobo-Dioulasso could be a risk factor for HPV infection in this population. Multiple infections with several HPV types are common among HIV infected women. In this study, women had little multiple infections (15.2%) with high-risk HPV compared to 90, 1% reported in HIV infected women by Zohoncon et al. [11]. The present study percentage is closer to the 12.3% of multiple infections reported in women attending gynecological consultation in Ouagadougou [10].

The results of this study could certainly not be generalized to the entire population of Bobo-Dioulasso but they helped to know the HPV genotypes present in a population of women in this city. This is of real significance in the sense that the data obtained in a city other than Ouagadougou will be used to have a broader overview of the distribution of high-risk HPV genotypes in Burkina Faso.

## 5. Conclusion

The most common genotypes in this study were HPV 39, HPV 52, HPV 18, and HPV 35. These results are consistent with those of other studies conducted in Burkina Faso, which showed that there was a predominance of high-risk HPV other than HPV 16 and HPV 18. The future vaccine trials should therefore consider these other high-risk genotypes in order to expand prevention through anti-HPV vaccination.

## Figures and Tables

**Table 1:** Sociodemographic, sexual, and behavioral characteristics of women included in the study.

Characteristics	Number	%
*Age groups in years*		
20–24	14	7.7
25–34	78	43.1
≥35	89	49.2
*Level of education*		
Illiterate	65	35.9
Primary	52	28.7
Secondary	62	34.3
University	02	1.1
*Marital status*		
Married or lives with a partner	152	84.0
Single	16	08.8
Widow	11	6.1
Divorced	02	1.1
*Number of sexual partners*		
0	13	7.2
1	164	90.6
≥2	04	2.2
*Occupation*		
Housewife	124	68.5
Pupil or student	13	7.2
Employee	16	8.8
Informal sector	28	15.5
*Use of contraception*		
No/natural method	120	66.3
Yes	61	33.7
*Age at the first sexual intercourse*		
13–17	48	30
18–24	109	68.1
≥25	03	1.9
Unanswered	21	
*Number of pregnancies*		
0	12	6,6
1-2	64	35.4
3–5	71	39.2
6–11	34	18.8
*Number of abortions*		
0	122	67.4
1	40	22.1
2	13	7.2
≥3	06	3.3

**Table 2:** HPV genotypes distribution according to the association with multiple or isolated infections and depending on the age.

HPV genotypes	Age < 30 *n* (%)	Age ≥ 30 *n* (%)	Total *n* (%)
*Genotypes associated with isolated infections *			
HPV 18	—	6 (13.0)	6 (13.0)
HPV 31	—	3 (6.5)	3 (6.5)
HPV 35	3 (6.5)	3 (6.5)	6 (13.0)
HPV 39	3 (6.5)	6 (13.0)	9 (16.7)
HPV 45	—	1 (2.2)	1 (2.2)
HPV 51	—	1 (2.2)	1 (2.2)
HPV 52	2 (4.4)	3 (6.5)	5 (10.9)
HPV 56	1 (2.2)	—	1 (2.2)
HPV 58	—	2 (4.3)	2 (4.4)
HPV 66	—	3 (6.5)	3 (6.5)
HPV 68	1 (2.2)	1 (2.2)	2 (4.4)

Total 1	10 (21.8)	29 (63.0)	39 (84.8)

*Genotypes associated with multiple infections *			
HPV 18+52	1 (2.2)	—	1 (2.2)
HPV 18+68	—	1 (2.2)	1 (2.2)
HPV 39+51	1 (2.2)	—	1 (2.2)
HPV 45+52	—	1 (2.2)	1 (2.2)
HPV 56+52	—	1 (2.2)	1 (2.2)
HPV 59+68	1 (2.2)	—	1 (2.2)
HPV 31+35+52	—	1 (2.2)	1 (2.2)

Total 2	3 (6.5)	4 (8.7)	7 (15.2)

General total	13 (28.3)	33 (71.7)	46 (100)

**Table 3:** Factors associated with oncogenic HPV infection.

	HPV− *n* = 135	HPV+ *n* = 46	Total *n* = 181	*p* ^*∗*^
*Age group*, *n (%)*				0.99
<30	40 (29.6)	13 (28.3)	53	
≥30	95 (70.4)	33 (71.7)	128	
*Age at first sexual intercourse, n (%)*				0.88
<16	12 (8.9)	05 (10.9)	17	
≥16	108 (80)	35 (76.1)	143	
Unanswered	15 (11.1)	06 (13.0)	21	
*Parity, n (%)*				0.76
Nulliparous	9 (6.7)	3 (6.5)	12	
Primiparous	18 (13.3)	7 (15.2)	25	
Multiparous	108 (80)	36 (78.3)	144	
*Use of oral contraception, n (%)*				0.09
Yes	16 (11.8)	01 (2.2)	17	
No	119 (88.2)	45 (97.8)	164	
*VIA/VILI result, n (%)* ^*∗∗*^				0.85
VIA/VILI−	127 (74.7)	43 (25.3)	170	
VIA/VILI+	7 (77.8)	2 (22.2)	9	

^*∗*^ *p* value for difference in characteristic according to HR-HPV status.

^*∗∗*^VIA/VILI result was available for *n* = 179 women.
